# Pollination networks along the sea-inland gradient reveal landscape patterns of keystone plant species

**DOI:** 10.1038/s41598-018-33652-z

**Published:** 2018-10-15

**Authors:** E. Fantinato, S. Del Vecchio, G. Silan, G. Buffa

**Affiliations:** Department of Environmental Sciences, Informatics and Statistics, Ca’ Foscari University of Venice, Via Torino 155, 30172 Venice, Italy

## Abstract

Linking the functional role of plants and pollinators in pollination networks to ecosystem functioning and resistance to perturbations can represent a valuable knowledge to implement sound conservation and monitoring programs. The aim of this study was to assess the resistance of pollination networks in coastal dune systems and to test whether pollination interactions have an explicit spatial configuration and whether this affect network resistance. To this aim, we placed six permanent 10 m-wide belt transects. Within each transect we placed five plots of 2 m x 2 m, in order to catch the different plant communities along the dune sequence. We monitored pollination interactions between plants and pollinators every 15 days during the overall flowering season. The resulting networks of pollination interactions showed a relatively low degree of resistance. However, they had a clear spatial configuration, with plant species differently contributing to the resistance of pollination networks occurring non-randomly from the seashore inland. Our results evidenced that beside contributing to the creation and maintenance of dune ridges, thereby protecting inland communities from environmental disturbance, plant species of drift line and shifting dune communities have also a crucial function in conferring resistance to coastal dune pollination networks.

## Introduction

Biodiversity loss is currently threatening the productivity and sustainability of Earth’s ecosystems^1^. According to Hooper *et al*.^2^, extinctions will accelerate ecosystem processes alteration, thereby compromising the functioning of ecosystems and affecting ecological services provided to humans. Even though biodiversity loss in the 21st century has been ranked among the major drivers of ecosystem change, little is still known on how the loss of biotic interactions triggered by local extinctions might affect the functioning of ecosystems^3^.

Among the wide variety of biotic interactions, animal-mediated pollination plays a decisive role in structuring natural communities^4,5^, by assuring plant reproductive success as well as pollinators maintenance^6^. Nowadays, mutualistic interactions for pollination are increasingly threatened giving rise to the “pollination crisis” phenomenon^7,8^. The implementation of conservation programs that aim to maintain interactions between plants and pollinators may manage to buffer and even reverse the local pollination crisis^9,10^. However, the effectiveness of conservation practices over time may be gained only if the ability of pollination interactions to withstand perturbations (e.g., species extinctions^11^) is safeguarded, as an assurance of the self-maintaining capacity of pollination networks.

Some topological patterns of the network structure have been shown to influence how pollination networks respond to ecological perturbations, that is, their ability to reduce the likelihood of perturbations or to be minimally affected by them, or the capacity to recover from them^12,13^. For example, general patterns of interactions such as redundancy, nestedness, asymmetry and modularity have been proven to confer stability by preventing the occurrence of secondary extinctions^13^. However, species’ network functional role can notably change across mutualistic partners, with different species contributing differently to the resistance of pollination networks^14^. Especially, highly generalist, partner-sharing species have been suggested to play a key role in the persistence of pollination interactions, by conferring resistance to the overall network of interactions, while, specialized and highly selective species occupy a peripheral position, contributing only marginally to the network stability^15^.

Linking network functional role of plant and pollinator species to ecosystem functioning represents a crucial goal for the conservation of natural and semi-natural communities; however, only recently studies on pollination networks have extended the focus to the landscape scale^16^. In fact, different plant communities can be deeply interconnected through flows of energy and materials at the landscape scale, and many examples of plant communities which naturally form continuous patterns in the landscape in response to environmental gradients have been described (e.g., river and lake edges, salt marshes).

Coastal dune systems are among the most outstanding examples worldwide of spatially connected plant communities^17,18^. Environmental disturbance in the form of salt spray, wind, soil movement and wave regime shapes coastal dunes selecting species according to their tolerance to the environmental disturbance and creating a precise sequence of ecologically distinct plant communities from the seashore inland, the so-called coastal zonation^19,20^. The coastal zonation reflects the progressively more sheltered environments that are created with increasing distance from the shoreline. Pioneer salt spray and sand blasting tolerant plant communities developing nearby the seashore are responsible for the formation and the building-up of dune ridges^21^ that act as a physical barrier to different stressors (e.g., wind, sand-blasting, waves), ultimately creating suitable conditions for the establishment of more disturbance-sensitive communities typical of semi-fixed and fixed dunes^22^.

Although the major dune-forming species are wind-pollinated grasses, animal-pollinated species occur in all plant communities of coastal dune systems and concur to the development of dune ridges (e.g., *Cakile maritima* Scop., *Echinophora spinosa* L., etc.^19^). Many of them are listed as diagnostic species for habitats definition in the Interpretation Manual of European Union Habitats (EU 28)^23^ and they are used as indicator species to define habitats’ conservation status^17,24^. Moreover, nowadays they are facing a huge decline because of coastal dune degradation and surface contraction^21,25^, and many of them have become a topic of increasing conservation concern^26,27^.

In the light of these considerations we assessed the function of pollination in coastal dune systems by answering the following questions: (i) does the richness of animal-pollinated plant and pollinator species show non-random spatial patterns from the seashore inland? (ii) Are coastal dune pollination networks resistant to perturbations (i.e., nested, modular and redundant) during the overall flowering season? (iii) Do plant species growing at different distances from the sea differently contribute to the resistance of coastal dune pollination networks?

## Material and Methods

### Study area

We selected an area along the North Adriatic coast (Italy), in the peninsula of Cavallino (45°26′22.14″N; 12°26′58.71″E), where a complete sequence of coastal dune communities, from the drift line to the inland fixed dune, can be observed. Sites consist of narrow, recent dunes (Holocene), that generally occupy a narrow strip along the seashore, bordered by river mouths and tidal inlets, mostly fixed by docks. The study area has a Temperate Oceanic bioclimate^28^. The annual average temperature is about 13 °C and the annual average precipitation is 831.5 mm^22^. Sediments are predominantly sandy carbonate deposits of dolomitic origin transported by river Piave and Tagliamento. The study area is under protection and has been subjected to conservation plans aimed at reducing natural erosion and human trampling^29^.

**Figure 1:**
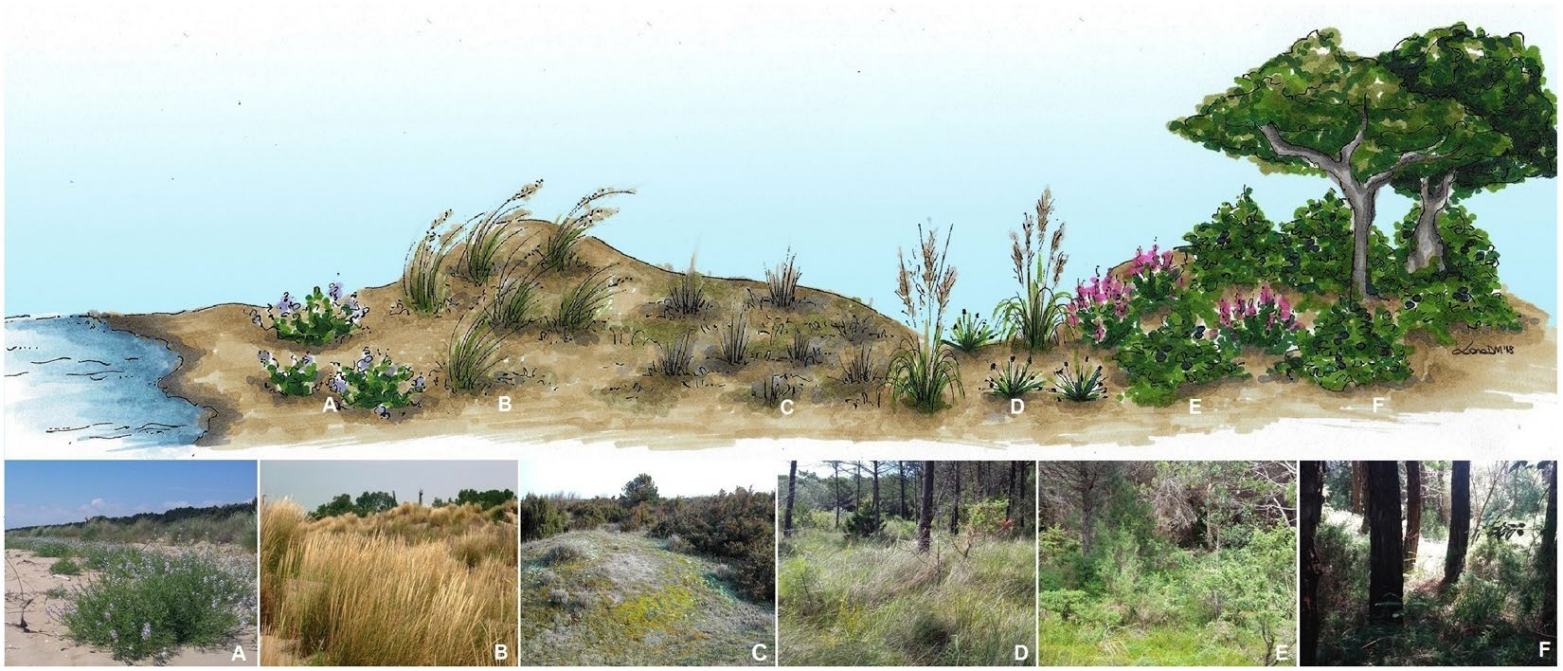
Simplified representation of the physiognomy of coastal dune systems along the North Adriatic Coast. Plant communities were identified by different letters: (**A**) pioneer community of the drift line, (**B**) pioneer community of shifting dunes, (**C**) xerophilous grasslands of semi-fixed dunes, (**D**) interdunal depressions, (**E**) xerophilous shrublands of fixed dunes, (**F**) xerophilous woodlands of fixed dune. We gratefully acknowledge Miss Lara Dal Molin for the hand painted picture.

In the study area, plant community sequence starts with the pioneer, nitrophilous community dominated by annuals of the drift line zone (*C. maritima* plant community). Being exposed to wave inundation, salt spray and wind stress, the community is often patchy and fragmented. Landwards, the *C. maritima* plant community is followed by the shifting dune community dominated by dune-forming plants such as *Elytrigia juncea* (L.) Nevski and *Ammophila arenaria* subsp. *australis* (Mabille) Laínz. *A. arenaria* subsp. *australis* is the dominant species and is responsible for stabilizing and building up the foredune by capturing blown sand and binding it together with its tough, fibrous rhizome system^30^. Inland of the foredune crest, protection from physical stresses increases and the vegetation evolves towards more structured forms. The coastal sequence includes perennial xerophilous grasslands of the semi-fixed dunes dominated by dwarf shrubs (e.g., *Fumana procumbens* (Dunal) Gren. & Godr., *Thymus pulegioides* L.), lichens (e.g., *Cladonia foliacea* (Huds.) Willd., *C. furcate* (Huds.) Schrad.) and mosses (e.g., *Syntrichia ruraliformis* (Besch.) Cardot), and interdunal depressions where a community of *Erianthus ravennae* (L.) H. Scholz and *Schoenus nigricans* L. can be observed. Lastly, the sequence ends with the xerophilous shrublands with *Erica carnea* L. and *Osyris alba* L. and the xerophilous woodlands with *Quercus ilex* L., *Pinus pinea* L. and *P. pinaster* Aiton of fixed dunes^31,32^ (Fig. 1).

### Data collection

At the beginning of the season we placed 6 permanent belt transects. Transects were 10 m wide and 606 ± 25 m (mean ± SD) long. The distance between transects was set at 50 m. Along each transect we identified plant communities and their spatial extent based on the habitat map of the Veneto region (available at https://www.regione.veneto.it/web/agricoltura-e-foreste/download#IT3250003; scale 1:10.000). Sampled communities were: (i) the pioneer community of the drift line, (ii) the pioneer community of shifting dunes, (iii) the xerophilous grasslands of semi-fixed dunes, (iv) the xerophilous shrublands of fixed dunes and (v) the xerophilous woodlands of fixed dunes (Fig. 1). All plant communities of the coastal system were sampled with the exception of interdunal depressions because of the almost total absence of animal-pollinated species. Within each transect we placed one plot of 2 m × 2 m per plant community, for a total of 5 plots per transect, and 6 replicates per plant community.

Due to the particular characteristics of coastal dune communities (e.g., scantily covering vegetation, prevalence of wind-pollinated species), within each 10 m wide belt transect and the spatial extent of each community, we placed the plots according to a preferential sampling design so as to catch at least one animal-pollinated species. Plots distance from the sea was measured through a trekking GPS (Garmin GPSmap 62 s). In each plot, plant species presence was recorded, and the flowering phenology of animal-pollinated species was monitored every fifteen days, from the beginning (April 03-04-2017) to the end of the flowering season (August 01-08-2017), for a total of nine surveys. The nine surveys were carried out in warm and sunny days^33,34^. Flowering monitoring started at the opening of the first flower and ended when individual plants no longer possessed any flower with anthers^35^. Furthermore, we quantified the length of flowering of each animal-pollinated species by counting the number of surveys in which a given species was observed flowering.

During each survey, we recorded visiting pollinators by counting the number of visits to each plant species. Each plot was monitored for 14 minutes, split into two 7-min sets distributed during two daily intervals (from 10 a.m. to 1 p.m. and from 1 p.m. to 4 p.m.) to ensure the observation of pollinators showing different daily periods of activity. We set the starting time of monitoring at 10 a.m. and the finishing time at 4 p.m., because, according to preliminary observations, pollination events were infrequent before 10 a.m. and after 4 p.m. Since literature reported nocturnal moths as potential pollinators of *Silene vulgaris* (Moench) Garcke^36^, we also conducted nocturnal surveys in order to observe pollination events mediated by nocturnal moths. However, no pollination contacts could be observed. Our methodology is in line with e.g., Lázaro *et al*.^33^, Hegland and Totland^37^ and Fantinato *et al*.^13^. In fact, with a few exceptions^38^, this time span has been proven to overlap the daily pattern of activity of the majority of pollinator species in temperate and Mediterranean ecosystems. In total we conducted 252 (i.e. 1,764 min) observation periods (126 in the morning and 126 in the afternoon) during the flowering season. We counted as pollinators only visitors landing on the flower, visiting it for more than 1 s, and getting in direct contact with the floral reproductive organs^37^. Both plants and pollinators were identified at the level of species or morphospecies. The identity of animal-pollinated plants and pollinators are included in the Electronic Supplementary Material 2.

### Sampling completeness

Since we sampled plant communities subjected to different environmental conditions, which might possibly lead to differences in pollinator activity, for each plant community we evaluated the sampling completeness of animal-pollinated plant and pollinator species, and of pollination interactions. We estimated the richness of (i) animal-pollinated plants, (ii) pollinators and (iii) pollination interactions with increased sampling effort, pooling data recorded in each plot during the overall flowering season. We computed the accumulation curves using the cumulative number of plots sampled per plant community as the unit of sampling effort and the Chao 2 estimator of asymptotic species richness^39^. We chose Chao 2 estimator, because it is one of the least biased estimates for a small number of samples^40–42^. Sampling completeness was calculated by quantifying the percentage of the asymptotic richness detected by the observed one^42^. The accumulation curves and the Chao 2 estimator were calculated with the R-based package *Vegan*^43^.

### Network parameters

For each survey, observed contacts were organized in an abundance matrix in which rows were plant species, columns were insect species, and entries represented the sum of all interactions observed between each plant and pollinator species. We chose to organize pollination interactions in one matrix for survey (for a total of 9 matrices) to avoid the formation of impossible interactions through pollinator sharing between plant species flowering during different surveys (i.e., forbidden links^44^). For each pollination matrix, we calculated the most used quantitative indices to describe the structure and resistance of weighted ecological interaction networks at the level of the entire dune system as well as at single species level. At the dune system level we calculated network selectiveness (H′_2_^45^), weighted nestedness (wNODF^46^) and quantitative modularity (Q^47^). A low degree of network selectiveness is linked to high interaction redundancy, which confers resistance to pollination networks by acting as a buffer against species loss. A nested pattern of interactions confers resistance to pollination networks by preventing the occurrence of secondary extinctions^48^, while a modular organization of interactions prevents the propagation of secondary extinctions through the pollination network^49^. The significance of observed results was tested by constructing 1000 randomized networks with identical margin totals as the empirical networks and comparing the observed and random values using the null model ‘r2d’^50^.

At the species level we chose indices revealing the contribution of each plant species to network selectiveness, nestedness and modularity. Especially, we calculated species selectiveness (d′^45^), which measures the exclusiveness of a species partner spectrum compared with other species in the network; nested contribution (n_i_^51^), which measures how individual species’ interactions change the degree of nestedness, calculated at the community level and compared to a random null model; weighted closeness centrality (wCC^52^), as a measure of the centrality of individual species within the topography of the network; standardized connection (c^14^) and participation values (z^14^), which measure the position of a species between modules and within its own module, respectively.

Network analysis were performed with R’s package “Bipartite” (R version 3.4.1; *bipartite* package version 2.08^53^).

### Data analysis

Since the extent of plant communities increased exponentially from the seashore inland, spanning from few meters for the pioneer community of the drift line to hundred meters for the coastal wood community, we log-transformed plot distance from the sea before data analysis.

Spatial variations in the richness of animal-pollinated plant and pollinator species were assessed through linear mixed models (LMMs; R-based package *nlme*^54^). We regressed the richness of animal-pollinated species in bloom with respect to a quadratic trend, while the richness of pollinators with respect to a linear trend. Especially, we included plot identity as random factor, the log-transformed distance from the sea as independent variable and the richness of (i) animal-pollinated species in bloom and (ii) pollinators as dependent. We used species richness of animal-pollinated species in bloom and pollinators recorded in each survey as replicates.

Subsequently, we calculated the mean log-transformed distance from the sea of each animal-pollinated species by averaging the log-transformed distance from the sea of the plots in which a given plant species was recorded. We assessed spatial variation of plant species selectiveness (d′), nested contribution (n_i_), weighted closeness centrality (wCC), standardized connection (c) and participation values (z) by means of linear mixed models (LMMs). We then regressed each index with respect to a linear trend, with the identity of species as random factor, the mean distance from the sea as independent variable and each single index as dependent. If a given plant species was observed to flower for more than one survey, we used values of indices calculated in each survey as replicates.

Furthermore, the spatial variation of the flowering length of animal-pollinated species was tested through a generalized linear model (GLM) using a Poisson distribution and log as link function with respect to a linear trend, the mean log-transformed distance from the sea as independent variable and the flowering length as dependent.

## Results

Overall, we recorded 521 contacts between 18 plants and 59 pollinators. The 18 animal-pollinated species belonged to 13 different families. The majority of plant families were represented by a single species (nine families). The most specious families were the Caryophyllaceae (three species), followed by the Fabaceae, Lamiaceae and Rosaceae (both with two species). Plant species were highly specialized for a single plant community; only few species were found in more than one plant community (e.g., *E. spinosa*, *Calystegia soldanella* (L.) Roem. & Schult.), and always with few individuals. The 59 pollinators recorded were all insects and belonged to six orders. Among the pollinator orders the most specious were the Hymenoptera (37 species), followed by the Coleoptera (eight species), Diptera and Lepidoptera (six species, both). Overall, 33 pollinator species were found in only one plant community, 15 species in two plant communities, six species in three plant communities, three species in four plant communities, while only *Apis mellifera* (Linnaeus, 1758) and *Bombus pascuorum* (Scopoli, 1763) were found in all plant communities.

**Figure 2:**
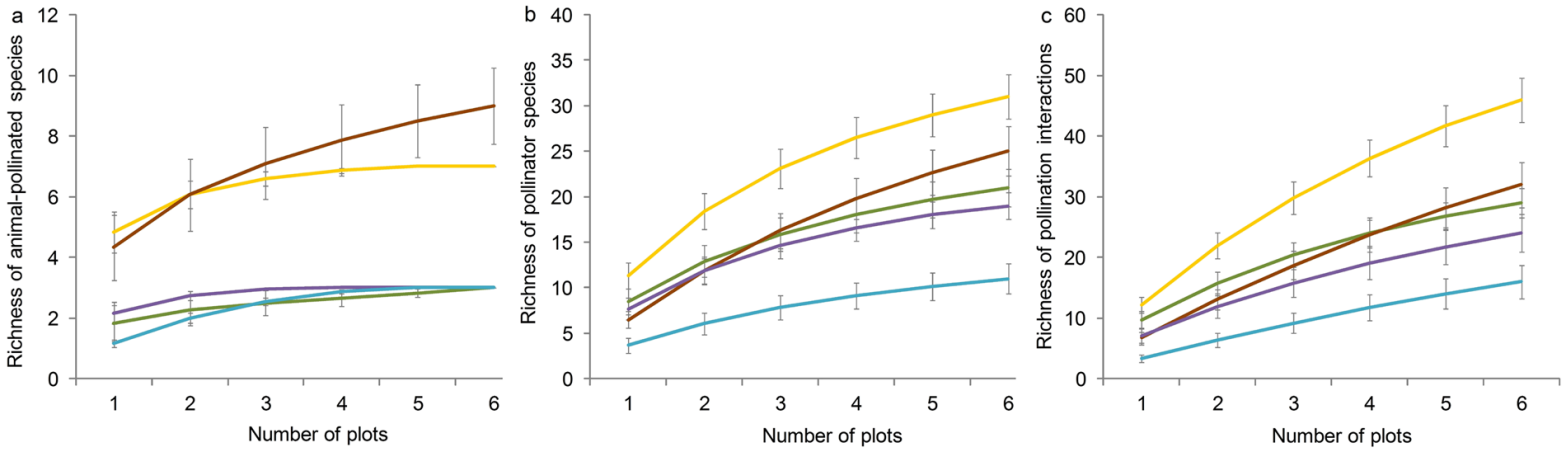
Accumulation curves of the richness of (**a**) animal-pollinated plants, (**b**) pollinators, and (**c**) pollination interactions. Accumulation curves were computed by using the cumulative number of plots sampled per plant community as the unit of sampling effort. Plant communities were represented by different colours: *green* pioneer community of the drift line, *yellow* pioneer community of shifting dunes, *red* xerophilous grasslands of semi-fixed dunes, *violet* xerophilous shrublands of fixed dunes, *blue* xerophilous woodlands of fixed dune.

Sampling completeness revealed that we detected more than 82.75% of animal-pollinated plants and more than 75.50% of pollinator species in all plant communities (Table 1; Fig. 2). For the richness of pollination interactions (Table 1; Fig. 2), detection varied among plant communities, diminishing from the pioneer community of the drift line (80.46% of pollination interactions detected) to the xerophilous woodlands of fixed dunes (51,61% of pollination interactions detected).

**Table 1:** Observed and asymptotic richness of (i) animal-pollinated plants, (ii) pollinators, and (iii) pollination interactions per plant community.

	Animal-pollinated species			Pollinator species			Pollination interactions		
	Observed richness	Asymptotic richness	Sampling completeness (%)	Observed richness	Asymptotic richness	Sampling completeness (%)	Observed richness	Asymptotic richness	Sampling completeness (%)
Pioneer community of the drift line	3.00	3.00	100.00	20.00	26.66	75.01	29.00	36.04	80.46
Pioneer community of shifting dunes	7.00	7.00	100.00	20.00	23.75	84.21	46.00	64.77	71.02
Xerophilous grasslands of semi-fixed dunes	9.00	10.87	82.79	18.00	20.55	87.59	32.00	56.49	56.64
Xerophilous shrublands of fixed dunes	3.00	3.00	100.00	14.00	17.47	80.13	24.00	40.33	59.50
Xerophilous woodlands of fixed dunes	3.00	3.00	100.00	9.00	10.25	87.80	16.00	31.00	51.61

Observed richness was calculated by pooling data recorded in each plot during the overall flowering season. Asymptotic richness was calculated using the Chao 2 estimator. Sampling completeness was calculated by quantifying the percentage of the asymptotic richness detected by the observed one.

**Figure 3:**
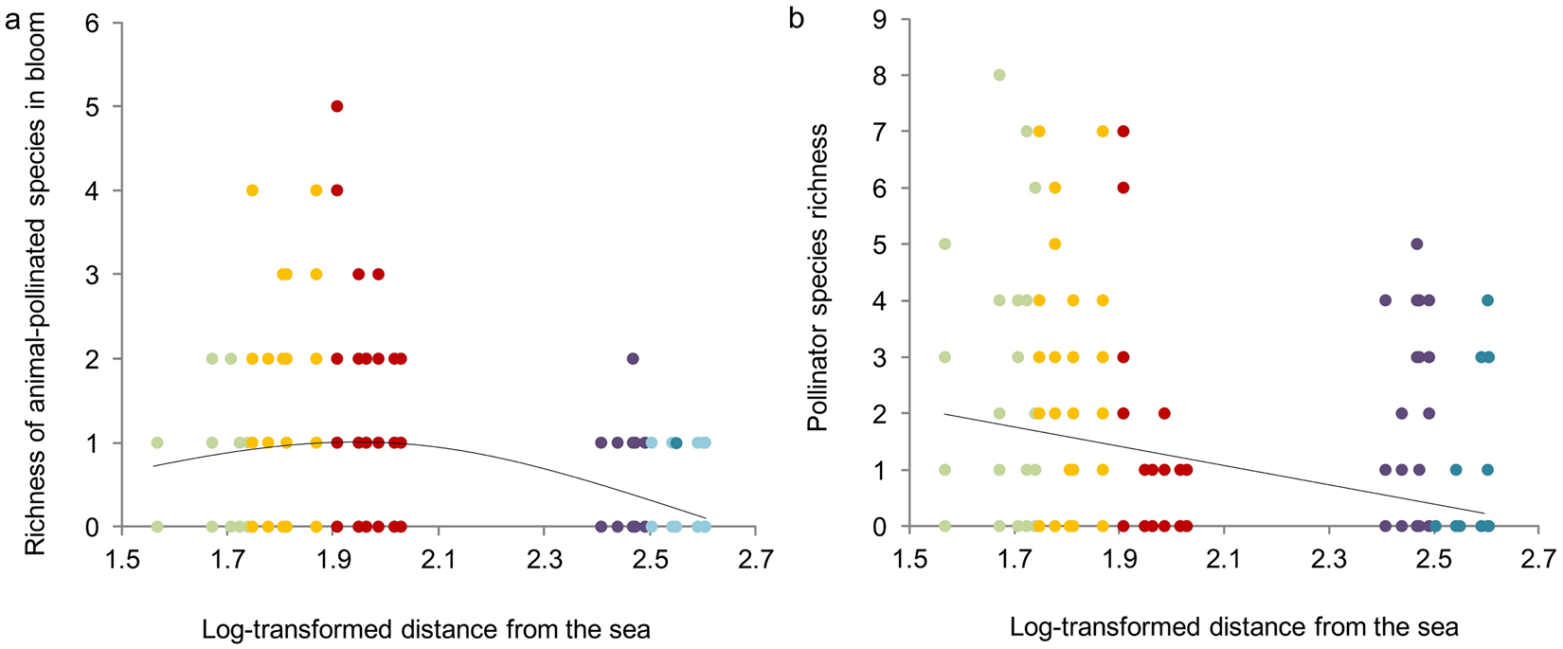
Linear Mixed Models (LMM) between plots log-transformed distance from the sea and the richness of (**a**) animal-pollinated plant and (**b**) pollinator species. Lines represent the estimates of the models. Dots of different colours represent different plant communities: *green* pioneer community of the drift line, *yellow* pioneer community of shifting dunes, *red* xerophilous grasslands of semi-fixed dunes, *violet* xerophilous shrublands of fixed dunes, *blue* xerophilous woodlands of fixed dune.

The richness of animal-pollinated species in bloom and of pollinator species notably changed along the sea-inland gradient. Linear mixed models (LLMs) revealed that the richness of animal-pollinated species in bloom followed a unimodal trend (t-value = −2.738; P = 0.011; Fig. 3a), with the peak of species richness coinciding with the xerophilous grasslands of semi-fixed dunes. On the contrary, pollinator species richness decreased significantly from the seashore inland, peaking in correspondence with the pioneer community of the drift lines (t-value = −3.227; P = 0.003; Fig. 3b).

Observed values of network selectiveness, nestedness and modularity were significantly different from random values throughout the flowering season (Table 2). Nevertheless, values of network selectiveness were always high (always >0.5), and coupled with low values of nestedness (<15; with the exception of the 9^th^ survey), and low values of modularity (almost always <0.6).

**Table 2:** Network parameters for each survey.

Survey	1st	2nd	3rd	4th	5th	6th	7th	8th	9th
H′_2_	1.000^***^	0.600^***^	0.780^***^	0.592^***^	0.601^***^	0.748^***^	0.801^***^	0.687^***^	0.681^***^
WNDOF	0.000^***^	14.102^***^	8.781^***^	9.190^***^	9.018^***^	4.991^***^	11.160^***^	9.484^***^	28.368^***^
Q	0.500^***^	0.347^*^	0.581^***^	0.521^***^	0.491^***^	0.662^***^	0.162^***^	0.502^***^	0.185^***^

H′_2_: network selectiveness; WNODF: weighted nestedness; Q: quantitative modularity. The significance of observed results was tested by constructing 1000 randomized networks with identical margin totals as the empirical networks and comparing the observed and random values using the null model ‘r2d’ (^*^P < 0.05; ^***^P < 0.001).

Linear mixed models (LMMs) revealed that species selectiveness (d′) significantly increased from the seashore inland, while weighted closeness centrality (wCC) and nestedness contribution (n_i_) significantly decreased (Table 3). The resulting networks of interactions had a clear spatial configuration, with plant species differently contributing to the organization of pollination interactions occurring non-randomly along the sea-inland gradient. Especially, plant species of the pioneer communities of the drift line and shifting dunes contributed more to the resistance of pollination interactions than plant species of the inland communities (i.e., xerophilous communities of semi-fixed dunes and fixed dunes). Plant species showing the lowest values of selectiveness (d′), coupled with the highest values of weighted closeness centrality (wCC) and nestedness contribution (n_i_) were *C. maritima*, *C. soldanella*, *E. spinosa* and *Medicago marina* L.

**Table 3:** Results of the linear mixed models (LMMs) comparing plant species level network indices and mean log-transformed distance from the sea.

	t-value	P
Species selectiveness (d′)	2.489	0.024
Nested contribution (n_i_)	−2.140	0.048
Weighted closeness centrality (WCC)	−2.279	0.037
Standardized connection (c)	−1.296	0.213
Participation value (z)	−0.516	0.615

Each index was regressed with respect to a linear trend, with the identity of species as random factor, the mean distance from the sea as independent variable and each single index as dependent. If a given plant species was observed to flower for more than one survey, we used values of indices calculated in each survey as replicates.

The flowering length of animal-pollinated species decreased significantly from the seashore inland (z-value = −2.249; P = 0.024), with *C. maritima* being the species flowering for the longest period of time in the studied system (6 surveys, approximately 2.5 months).

## Discussion

Coastal dune systems are shaped by strong environmental gradients which determine the co-occurrence, in a relatively small area, of different plant communities. The steep gradient allows the presence of communities characterized by a high ecological diversity in terms of environmental properties as well as of species composition, with different pools of plant and pollinator species showing high specialization for a single plant community. In terms of richness of animal-pollinated species, our results confirm xerophilous grasslands of semi-fixed dunes as the most outstanding community of costal dune systems. This pattern was already evidenced by previous studies^19^ which observed an increase in plant species richness following the sea-inland gradient, with a slight decrease in the woody community of fixed dunes. Accordingly, richness of animal-pollinated species followed a unimodal trend, reaching the highest values in correspondence with the xerophilous grasslands of semi-fixed dunes. Being less exposed to the environmental disturbance, xerophilous grasslands normally present a higher plant species richness compared to both the pioneer communities of drift line and shifting dunes^19,20^. Interestingly, pollinator species richness did not follow a comparable trend; pollinator richness significantly decreased from the seashore inland, with a peak in the pioneer community of drift lines, which, however, hosts a low number of animal-pollinated species.

In coastal dune systems, few animal-pollinated species (i.e., *C. maritima*, *C. soldanella*, *E. spinosa*, *M*. *marina*) are responsible for the maintenance of pollinator species richness. The observed pattern is in contrast with those observed in other plant communities^55,56^. Numerous empirical studies report a positive relationship between the richness of plant species and that of pollinator species^57,58^. The positive impact of the plant species richness on the richness of pollinator species has been explained by increasing floral resource heterogeneity (nectar and pollen), which increases attractiveness for many pollinator species seeking single and multiple resources^8^. However, it is possible that species with large flowers/inflorescences like *C. maritima*, *C. soldanella*, *E. spinosa* and *M. marina*, attract more pollinators than plant species with isolated, often poorly attractive flowers/inflorescences like, e.g. *Cerastium semidecandrum* L., *F. procumbens* and *S. vulgaris*, which are typical of the xerophilous communities of the semi-fixed dunes (but see Ebeling *et al*.^56^). In this context, the quantification of floral resources (nectar and pollen) produced by each species may provide new insights into the observed mismatch between the richness of plant and pollinator species, thus revealing how few animal-pollinated species can sustain the richness of pollinator species in coastal dune systems.

Another explanation might be related to an unbalanced sampling effort among plant communities. However, our analysis of sampling completeness revealed that the sampling effort allowed us to catch a high proportion of the richness of animal-pollinated plant and pollinator species in all plant communities. A decreasing completeness from the pioneer community of the drift line to the xerophilous woodlands of fixed dunes was detected only for pollination interactions. One possible explanation is that, moving inland, pollination interactions become more selective, resulting in a higher probability to observe single interactions. Chacoff *et al*.^42^ pointed out that, since the Chao 2 estimator quantifies completeness on the basis of infrequent species (or interactions)^40,41^, a difference between the detected number of plant and pollinator species, and that of pollination interactions, could be attributed to the presence of selective interactions, rather than to true undersampling of interactions. However, this remains a point that deserves further studies and we cannot exclude that a further sampling effort might have increased the completeness of pollination interactions in the xerophilous communities of semi-fixed and fixed dunes.

Interestingly, in accordance with the decreasing see-inland trend of completeness of pollination interactions, we found that the degree of selectiveness (d′) of plant species increased throughout the coastal dune system, reaching the highest values in the xerophilous woodlands of fixed dunes. High selectiveness entails a high dependence of plant species to pollinator species, thereby resulting in less resistance to perturbations. Conversely, a low plant selectiveness is associated with high pollinator sharing and thus redundancy of interactions. Redundant interactions confer high resistance to pollination networks, acting as a buffer against species loss. Beside the increasing trend of plant species selectiveness from the seashore inland, we found a significant negative correlation between values of weighted closeness centrality (wCC), as well as of nestedness contribution (n_i_), and the mean log-transformed distance from the sea. Animal-pollinated species growing in the pioneer communities of drift line and shifting dunes were more central in the topography of pollination networks (i.e., keystone species^47^) than those occurring more inland (i.e., peripheral species^47^). According to Martin González *et al*.^15^ the removal of keystone plant species would have the strongest effects on a pollination network and might even lead to its collapse by triggering the local secondary extinction of specialized pollinator species.

Coastal dune pollination networks were non-random during the overall flowering season. Among network parameters, a non-random nested organization of pollination interactions is expected to increase system resistance by decreasing the probability of local secondary extinction of specialists, which are considered the most vulnerable network members^59^. Furthermore, a non-random modular organization of pollination interactions has been recognized to slow the spread of disturbances, further increasing the resistance of pollination networks^60^. However, values of network selectiveness, nestedness and modularity revealed that the resistance of coastal dune pollination networks was relatively low when compared with pollination networks assessed in other plant communities^52,61,62^. Such relatively low resistance of coastal dune pollination networks further confirms the crucial role of plant species of drift line and shifting dune communities for the prevention of pollination network collapse.

Observed non-random sea-inland pattern of pollination interactions might be the result of the selective pressure exerted by the environmental disturbance on animal-pollinated species. Indeed, according to the specialization-asymmetry-disturbance hypothesis, both specialization and asymmetry of interactions (i.e., nested pattern of interactions^63^) would explain species’ responses to disturbance^64^. In support to this theory, we showed that plant species selectiveness (d′) increased along the sea-inland gradient, while their nestedness contribution (n_i_) decreased. In fact, the environmental disturbance to which drift line and shifting dune communities are subjected may increase the stochasticity of pollination interactions, mostly affecting specialized, highly selective, species^6^. Moreover, we proved that animal-pollinated species growing in proximity of the seashore flowered for a longer period than those occurring in the more inland plant communities. A longer flowering period may, in fact, increase the chances of pollination also under variable environmental conditions and, in turn, assure stability to pollination networks during the overall flowering season^65^.

In conclusion, despite some criticalities, our results provided new insights into pollination interactions along environmental gradients. The decreased completeness of pollination interactions from the seashore inland should be further investigated, to disentangle the effect of undersampling from the spatial pattern of species selectiveness. Furthermore, a trait-based approach, aimed at revealing the sea-inland gradient of floral resources, may improve our understanding of the observed spatial pattern of pollinator species richness.

Nevertheless, our study proved that, when different plant communities are spatially interconnected, the assessment of pollination interactions at the landscape scale results in a better understanding of ecosystem dynamics and resistance to perturbations. In the case of coastal dune systems, our results highlighted the essential function of plant species growing nearby the seashore in conferring resistance to pollination networks. Thus, the key function of plant species of drift line and shifting dune communities is not limited only to the creation and maintenance of dune ridges^19^, but also to the maintenance of the stability of pollination interactions over the flowering season.

In order to increase the effectiveness of conservation practices in coastal dune systems, it will be crucial to consider the structure of pollination interactions at the landscape scale along the sea-inland gradient. Focusing on the key role of plant species of the drift line and shifting dune communities would assure resistance, and thus stability, to coastal dune pollination networks, ultimately increasing the long-term sustainability of conservation actions.

## Electronic supplementary material

Supplementary Material 1

Supplementary Material 2

## Data Availability

Data analysed during this study are included in the Electronic Supplementary Material 2.

## References

[1] Cardinale BJ (2012). Biodiversity loss and its impact on humanity. Nature.

[2] Hooper DU (2012). A global synthesis reveals biodiversity loss as a major driver of ecosystem change. Nature.

[3] Kaiser–Bunbury CN, Blüthgen N (2015). Integrating network ecology with applied conservation: a synthesis and guide to implementation. AoB Plants.

[4] Fantinato E, Giovanetti M, Del Vecchio S, Buffa G (2016). Altitudinal patterns of floral morphologies in dry calcareous grasslands. Plant Sociol..

[5] Fantinato E, Del Vecchio S, Giovanetti M, Acosta ATR, Buffa G (2018). New insights into plants coexistence in species–rich communities: the pollination interaction perspective. J. Veg. Sci..

[6] Dante SK, Schamp BS, Aarssen LW (2013). Evidence of deterministic assembly according to flowering time in an old–field plant community. Funct. Ecol..

[7] Memmott J, Waser NM, Price MV (2004). Tolerance of pollination networks to species extinctions. Proc. R. Soc. B Biol. Sci..

[8] Ghazoul J (2006). Floral diversity and the facilitation of pollination. J. Ecol..

[9] Haaland C, Naisbit RE, Bersier LF (2011). Sown wildflower strips for insect conservation: A review. Insect. Conserv. Divers..

[10] Feltham H, Park K, Minderman J, Goulson D (2015). Experimental evidence that wildflower strips increase pollinator visits to crops. Ecol. Evol..

[11] Elle E, Elwell SL, Gielens GA (2012). The use of pollination networks in conversation. Botany.

[12] Welti EAR, Joern A (2018). Fire and grazing modulate the structure and resistance of plant–floral visitor networks in a tallgrass prairie. Oecologia.

[13] Fantinato, E., Del Vecchio, S., Gaetan, C. & Buffa, G. The resilience of pollination interactions: importance of temporal phases. *J. Plant Ecol*, 10.1093/jpe/rty005 (2018).

[14] Olesen JM, Bascompte J, Dupont YL, Jordano P (2007). The modularity of pollination networks. Proc. Natl. Acad. Sci. USA.

[15] Martín González AM, Dalsgaard B, Olesen JM (2010). Centrality measures and the importance of generalist species in pollination networks. Ecol. Complex..

[16] Moreira EF, Boscolo D, Viana BF (2015). Spatial heterogeneity regulates plant–pollinator networks across multiple landscape scales. PLoS One.

[17] Del Vecchio, S., Slaviero, A., Fantinato, E. & Buffa, G. The use of plant community attributes to detect habitat quality in coastal environments. *AoB Plants***plw8** (2016). 10.1093/aobpla/plw040 PMC4940507 27255516

[18] Sperandii MG, Prisco I, Acosta ATR (2018). Hard times for Italian coastal dunes: insights from a diachronic analysis based on random plots. Biodiv. Conserv..

[19] Acosta A, Carranza ML, Izzi CF (2009). Are there habitats that contribute best to plant species diversity in coastal dunes. Biodiv. Conserv..

[20] Silan G, Del Vecchio S, Fantinato E, Buffa G (2017). Habitat quality assessment through a multifaceted approach: The case of the habitat 2130^∗^ in Italy. Plant. Sociol..

[21] Martínez, M. L., Psuty, N. P. & Lubke, R. A. A perspective on coastal dunes(ed. Martínez, M. L. & Psuty, N. P.) 171, 3–10 (Coastal dunes. Ecology and Conservation. EcologicalStudies, Springer, Berlin 2004).

[22] Buffa, G., Fantinato, E. & Pizzo, L. (ed. Lameed, G. A.) Effects of disturbance on sandy coastal ecosystems of N–Adriatic coasts (Italy) 339–372 (Biodiversity Enrichment in a Diverse World).

[23] EEC. Interpretation Manual of European Union Habitats – EUR28. European Commission DG Environment, Nature ENV B.3 (2013).

[24] Del Vecchio S, Pizzo L, Buffa G (2015). The response of plant community diversity to alien invasion: Evidence from a sand dune time series. Biodiv. Conserv..

[25] Janssen, J. A. M. *et al*. European Red List of Habitats – Part 2 (Terrestrial and Freshwater Habitats, 2016).

[26] Calvão T, Pessoa M, Lidon F (2013). Impact of human activities on coastal vegetation - a review. Emir. J. Food Agric..

[27] IUCN, The IUCN Red List of Threatened Species Version 2017–3, http://www.iucnredlistorg (2017).

[28] Del Vecchio S (2018). Biogeographic variability of coastal perennial grasslands at the European scale. Appl. Veg. Sci..

[29] Bezzi A, Fontolan G, Nordstrom KF, Carrer D, Jackson NL (2009). Beach nourishment and foredune restoration: practices and constraints along the Venetian shoreline, Italy. J. Coast. Res..

[30] Maun, M. A. The biology of coastal sand dunes (Oxford, UK: Oxford University Press 2009).

[31] Sburlino G, Buffa G, Filesi L, Gamper U, Ghirelli L (2013). Phytocoenotic diversity of the N–Adriatic coastal sand dunes – The herbaceous communities of the fixed dunes and the vegetation of the interdunal wetlands. Plant Sociol..

[32] Gamper U, Filesi L, Buffa G, Sburlino G (2008). Diversità fitocenotica delle dune costiere nord–adriatiche. Plant Byosist..

[33] Lázaro A (2016). Moderation is best: effects of grazing intensity on plant-flower visitor networks in Mediterranean communities. Ecol. Appl..

[34] Fantinato E, Del Vecchio S, Baltieri M, Fabris B, Buffa G (2017). Are food–deceptive orchid species really functionally specialized for pollinators?. Ecol. Res..

[35] Fantinato E (2016). Does flowering synchrony contribute to the sustainment of dry grassland biodiversity?. Flora – Morphol. Distrib. Funct. Ecol. Plants.

[36] Kephart S, Reynolds RJ, Rutter MT, Fenster CB, Dudash MR (2006). Pollination and seed predation by moths on Silene and allied Caryophyllaceae: evaluating a model system to study the evolution of mutualisms. New Phytol..

[37] Hegland SJ, Totland Ø (2005). Relationships between species’ floral traits and pollinator visitation in a temperate grassland. Oecologia.

[38] Herrera CM (1990). Daily patterns of pollinator activity, differential pollinating effectiveness, and floral resource availability, in a summer-flowering Mediterranean shrub. Oikos.

[39] Colwell RK, Chang XM, Chang J (2004). Interpolating, extrapolating, and comparing incidence-based species accumulation curves. Ecology.

[40] Colwell RK, Coddington JA (1994). Estimating terrestrial biodiversity through extrapolation. Philos. Trans. R. Soc. Lond., B, Biol. Sci..

[41] Chao A, Colwell RK, Lin C-W, Gotelli NJ (2009). Sufficient sampling for asymptotic minimum species richness estimators. Ecology.

[42] Chacoff NP (2012). Evaluating sampling completeness in a desert plant-pollinator network. J. Anim. Ecol..

[43] Oksanen, J. *et al*. Vegan: Community Ecology Package. R package version 2.0-9. http://CRAN.R-project.org/package=vegan (2013).

[44] Jordano P, Bascompte J, Olesen MJ (2003). Invariant Properties in Coevolutionary Networks of Plant – Animal Interactions. Ecol. Lett..

[45] Blüthgen N, Menzel F, Blüthgen N (2006). Measuring specialization in species interaction networks. BMC Ecol..

[46] Galeano J, Pastor JM, Iriondo JM (2009). Weighted–Interaction Nestedness Estimator (WINE): A new estimator to calculate over frequency matrices. Environ. Modell. Softw..

[47] Dormann CF, Strauss R (2014). A method for detecting modules in quantitative bipartite networks. Methods Ecol. Evol..

[48] Traveset A, Castro–Urgal R, Rotllàn–Puig X, Lázaro A (2018). Effects of habitat loss on the plant–flower visitor network structure of a dune community. Oikos.

[49] Tylianakis JM, Laliberté E, Nielsen A, Bascompte J (2010). Conservation of species interaction networks. Biol. Conserv..

[50] Guimerà R, Amaral LAN (2005). Functional cartography of complex metabolic networks. Nature.

[51] Saavedra S (2011). Strong contributors to network persistence are the most vulnerable to extinction. Nature.

[52] Ballantyne G, Baldock KCR, Willmer PG (2015). Constructing more informative plant–pollinator networks: visitation and pollen deposition networks in a heathland plant community. Proc. R. Soc. B Biol. Sci..

[53] Dormann CF, Gruber B, Fründ J (2008). Introducing the bipartite Package: analysing ecological networks. R news.

[54] Pinheiro, J., Bates, D., DebRoy, S., Sarkar, D. & R Core Team. nlme: Linear and Nonlinear Mixed Effects Models. R package version 3.1-120, http://CRAN.R-project.org/packa-ge=nlme (2015).

[55] Fontaine C, Dajoz I, Meriguet J, Loreau M (2006). Functional diversity of plant–pollinator interaction webs enhances the persistence of plant communities. PLoS Biol.

[56] Ebeling A, Klein AM, Schumacher J, Weisser WW, Tscharntke T (2008). How does plant richness affect pollinator richness and temporal stability of flower visits?. Oikos.

[57] Holzschuh A, Steffan‐Dewenter I, Kleijn D, Tscharntke T (2007). Diversity of flower‐visiting bees in cereal fields: effects of farming system, landscape composition and regional context. J. Appl. Ecol..

[58] Sárospataki M (2009). Factors affecting the structure of bee assemblages in extensively and intensively grazed grasslands in Hungary. Community Ecol..

[59] Joppa LN, Montoya JM, Solé R, Sanderson J, Pimm SL (2010). On nestedness in ecological networks. Evol. Ecol. Res..

[60] Krause AE, Frank KA, Mason DM, Ulanowicz RE, Taylor WW (2003). Compartments revealed in food- web structure. Nature.

[61] Traveset A, Chamorro S, Olesen JM, Heleno R (2015). Space, time and aliens: charting the dynamic structure of Galápagos pollination networks. AoB PLANTS.

[62] Gonzalez O, Loiselle BA (2016). Species interactions in an Andean bird–flowering plant network: phenology is more important than abundance or morphology. PeerJ.

[63] Vázquez, D. P. & Aizen, M. A. Community–wide patterns of specialization in plant–pollinator interactions revealed by null models (ed. Waser, N. M. & Ollerton, J.) 200–219 (Plant–Pollinator Interactions: from Specialization to Generalization. Univ Chicago Press, Chicago 2006).

[64] Vázquez DP, Simberloff D (2002). Ecological specialization and susceptibility to disturbance: conjectures and refutations. Am. Nat..

[65] Elzinga JA (2007). Time after time: flowering phenology and biotic interactions. Trends Ecol. Evol..

