# Supplementary material for: Pollination networks along the sea-inland gradient reveal landscape patterns of keystone plant species
Source: Sci Rep. 2018 Oct 15;8:15221. doi: 10.1038/s41598-018-33652-z (PMC6189214; doi:10.1038/s41598-018-33652-z)
Supplement: Supplementary file 1 — Supplementary Material 1 [file 41598_2018_33652_MOESM1_ESM.pdf]

Supplementary material 1 - Map of the study area

Pollination networks along the sea-inland gradient reveal landscape patterns of keystone plant species

Fantinato E.*, Del Vecchio S., Silan G., Buffa G.

Department of Environmental Sciences, Informatics and Statistics, Ca' Foscari University of Venice, Via Torino 155, 30172 Venice, Italy

*Corresponding author
E-mail address: edy.fantinato@unive.it
Telephone number: +39 041 234 7741

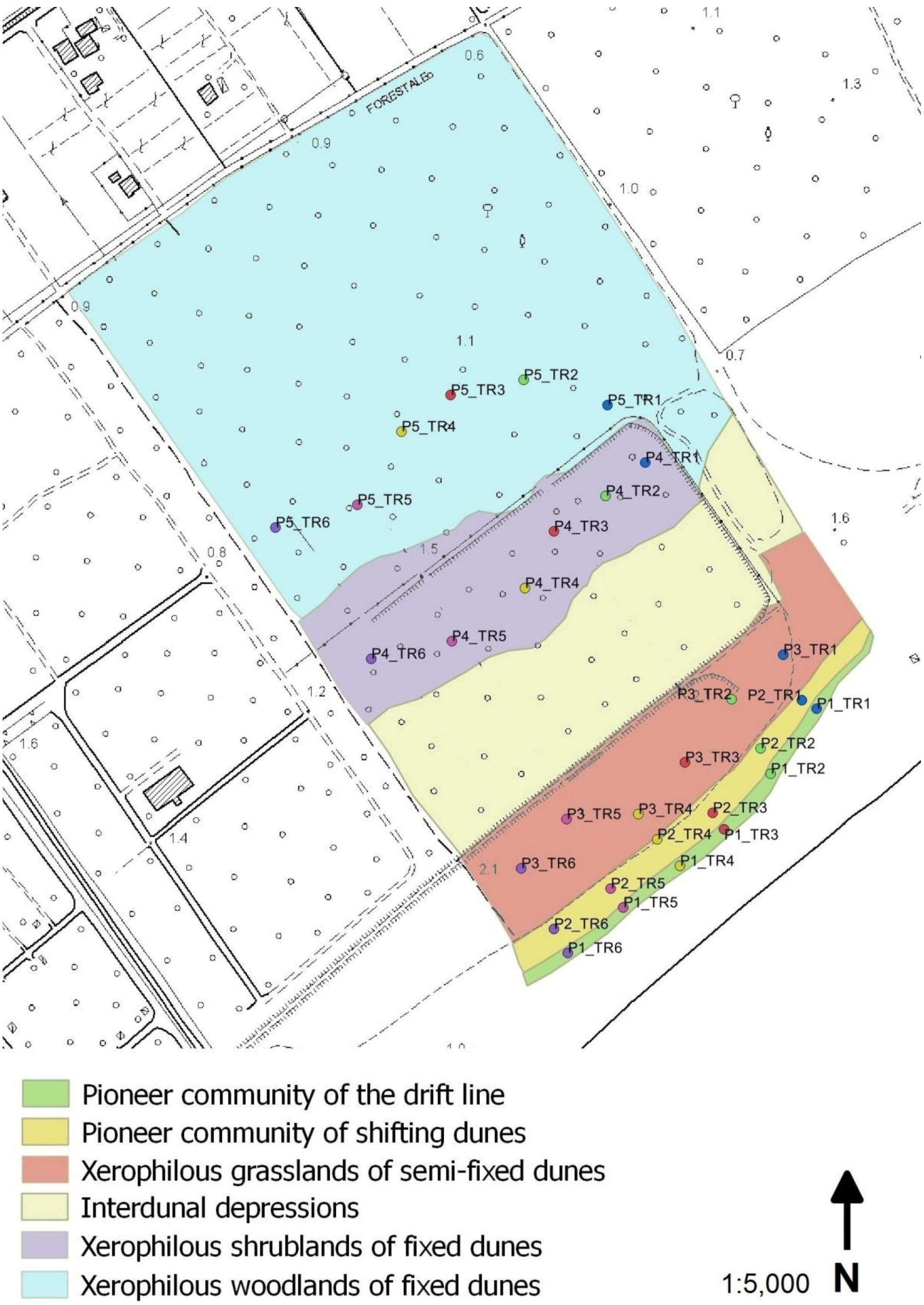

Map of the study area. Plots belonging to different transects are represented by different colors. Plots' label includes two information; *P*, followed by the number of the plot, and *TR*, followed by the number of the transect. Background layers include: modified habitat map of the Veneto region (available at https://www.regione.veneto.it/web/agricoltura-e-foreste/download#IT3250003; scale 1:10.000), and Veneto Regional Technical Map.
